# Bone Morphogenetic Protein-10 as a Predictor of Atrial Fibrillation Recurrence After Catheter Ablation: A Systematic Review and Meta-Analysis

**DOI:** 10.14740/jocmr6640

**Published:** 2026-09-26

**Authors:** Naveed Mohsin, Martin Eugene Matsumura, Tariq Ahmad, Kaif Ul Sahar

**Affiliations:** a Department of Internal Medicine, Geisinger Wyoming Valley Hospital, Wilkes-Barre, PA 18711, USA; b Department of Cardiology, Geisinger Wyoming Valley Hospital, Wilkes-Barre, PA 18711, USA; c Department of Sciences, University of Kashmir, Srinagar, India

**Keywords:** Bone morphogenetic protein-10, Catheter ablation, Atrial fibrillation recurrence

## Abstract

**Background:**

Biomarkers of atrial cardiomyopathy may improve the prediction of recurrence of atrial fibrillation (AF) after catheter ablation. Bone morphogenetic protein-10 (BMP10) is an atrial-specific cardiomyocyte protein and a potential biomarker, but evidence is limited and not comprehensively synthesized.

**Methods:**

We performed a systematic review and meta-analysis of prospective cohort studies to evaluate the association between circulating BMP10 concentrations with AF recurrence after catheter ablation. Pooled hazard ratios (HRs) with corresponding 95% confidence intervals (CIs) were computed using a random-effects model. The primary analysis used unadjusted HRs per continuous BMP10 levels from all included studies. Secondary analyses were conducted based on the adjusted HRs of studies with continuous BMP10 and all studies, including one study that reported adjusted HRs based on quartiles.

**Results:**

Three studies were included. In the primary analysis without adjustment (n = 3), increased levels of BMP10 were significantly associated with recurrence of AF (pooled HR 1.53, 95% CI 1.13–2.07; P = 0.006), with moderate-to-high heterogeneity (I^2^ = 69%). The association was attenuated and non-significant in the adjusted analysis of two studies with continuous BMP10 (HR 1.46, 95% CI 0.94–2.25; I^2^ = 59%). However, after the inclusion of a third study with adjusted quartile-based HR, the statistical significance was re-demonstrated (HR 1.33, 95% CI 1.16–1.53; P < 0.001) with low heterogeneity (I^2^ = 20%).

**Conclusion:**

Elevated BMP10 levels are correlated with higher risk of AF recurrence following catheter ablation. Although the results are consistent across analyses, they should be interpreted with caution due to the small number of studies and methodological heterogeneity. BMP10 needs to be confirmed as a clinically applicable biomarker by larger prospective studies.

## Introduction

Atrial fibrillation (AF) is the most common supraventricular arrhythmia, affecting approximately 37.6 million people worldwide, accounting for nearly 0.5% of the global population, and the incidence is steadily increasing [1]. AF is associated with a markedly increased risk of stroke, heart failure, sudden cardiac death, and impaired quality of life. Older adults are especially vulnerable and account for a disproportionate burden of morbidity, mortality, and disability from AF [2].

Early rhythm control has become an important part of contemporary AF management [3–5]. In addition to the improvement in the symptoms related to AF [6], rhythm control therapy reduces major cardiovascular outcomes such as stroke, heart failure hospitalizations, and cardiovascular mortality [3, 7]. The EAST-AFNET-4 trial showed that the cardiovascular benefit of early rhythm control is mainly mediated by the attainment and maintenance of sinus rhythm at 12 months [4], which may reflect a lower AF burden [8] and less progression to persistent or permanent AF [9].

Catheter ablation (CA) is an-established rhythm control strategy and a safe and effective alternative to antiarrhythmic drug therapy for maintenance of sinus rhythm [10, 11]. Notwithstanding the improvements in ablation technologies and techniques, AF recurs in about one-third of patients after CA and still remains a major clinical challenge [12]. The mechanisms underlying AF recurrence after ablation are not completely understood [13], and reliable prediction of recurrence in routine clinical practice is limited [14].

Proposed mechanisms of post-ablation AF recurrence include atrial cardiomyocyte loss, atrial stretch, inflammation, fibrosis, metabolic dysregulation, endothelial dysfunction, and altered cellular proliferation [13]. Accurate identification of patients at increased risk of recurrence is essential to guide therapeutic decision making; however, current risk stratification approaches rely predominantly on clinical variables [11, 15].

Several post-ablation clinical risk scores have been developed to predict AF recurrence, but recent systematic reviews and meta-analyses show modest discrimination performance and limited clinical utility [14, 16]. Consequently, increasing attention has been directed toward integrating circulating biomarkers with established clinical, imaging, and procedural variables to improve individualized risk assessment [17]. Rather than replacing existing prediction models, biomarkers such as bone morphogenetic protein (BMP) may provide complementary information reflecting the underlying extent of atrial remodeling and disease severity.

Atrial remodeling with collagen deposition and fibrosis is closely associated with AF pathogenesis [18] and recurrence after CA [19]. Bone morphogenetic protein-10 (BMP10), a member of the transforming growth factor-β superfamily, has been implicated in cardiac remodeling [20]. BMP10 is an atrial-specific circulating biomarker, regulated by PITX2 [20], and released into the circulation during atrial development and structural remodeling [21]. Previous studies have shown association of BMP10 levels with AF recurrence after CA [22], and adverse cardiovascular outcomes in patients with AF, independent of oral anticoagulation [23].

However, the evidence so far is limited by heterogeneity in study design and small sample sizes. Therefore, the objective of this systematic review and meta-analysis is to assess the role of BMP10 as a predictor of AF recurrence after catheter ablation, in order to improve pre-ablation risk stratification and guide clinical decision making in patients at high risk of recurrence.

## Materials and Methods

This systematic review and meta-analysis was conducted in accordance with Preferred Reporting Items for Systematic Reviews and Meta-analysis (PRISMA) 2020 guidelines [24]. Eligible studies were identified from major databases using search strategy, eligibility criteria, and quality appraisal tools. A random-effects primary meta-analysis was performed with assessment of heterogeneity, and secondary analyses was performed to check the robustness of association, as described below.

### Search strategy and databases

To identify relevant articles, a comprehensive literature search was conducted since inception through February 2026 on PubMed/Medline (using Medical Subject Headings (MeSH)), Cochrane Library, and Europe PubMed Central. On two other databases (Science Direct and Google Scholar), literature search was done from January 2015 through February 2026, and supplemented by scanning through bibliographies of articles. The search strategy was developed using keywords such as “Bone morphogenetic protein 10 (BMP-10), atrial fibrillation recurrences, and catheter ablation”. A more detailed account of the search strategies, and the number of studies identified can be found in Table 1.

**Table 1:** Search Strategies on Major Databases

Database	Keywords/search strategy	Filters	Search result
Pubmed MeSH/Medline	"BMP-10"[All Fields] OR "Bone morphogenetic protein 10"[Text Word] OR "Bone Morphogenetic Proteins"[MeSH Terms]) AND ("Atrial fibrillation recurrence"[Text Word] OR "atrial fibrillation"[Text Word] OR ("recurrance"[All Fields] OR "Recurrence"[MeSH Terms] OR "Recurrence"[All Fields] OR "recurrences"[All Fields] OR "recurrencies"[All Fields] OR "recurrency"[All Fields] OR "recurrent"[All Fields] OR "recurrently"[All Fields] OR "recurrents"[All Fields]) OR "AF free period"[Text Word] OR "atrial fibrillation"[MeSH Terms] OR "Recurrence"[MeSH Terms]) AND ("catheter ablation"[Text Word] OR ("cryoablated"[All Fields] OR "Cryosurgery"[MeSH Terms] OR "Cryosurgery"[All Fields] OR "cryoablation"[All Fields] OR "cryoablations"[All Fields]) OR "Radiofrequency catheter ablation"[Text Word] OR "pulmonary vein* isolation"[Text Word] OR "catheter ablation"[MeSH Terms] OR "Cryosurgery"[MeSH Terms] OR "Radiofrequency Ablation"[MeSH Terms]	Filters applied: English, humans, exclude preprints.	4
Cochrane Library	"BMP-10" OR "Bone morphogenetic protein 10" AND "Atrial fibrillation recurrence" AND “Catheter ablation”		2
Science Direct	"BMP-10" OR "Bone morphogenetic protein 10" AND "Atrial fibrillation recurrence" AND "Catheter ablation"	Last 10 years, research/review articles, medicine & dentistry, English, open access and open archive	16
Europe PubMed central	"BMP-10" OR "Bone morphogenetic protein 10" AND "Atrial fibrillation recurrence" AND "Catheter ablation" OR cryoablation OR "Radiofrequency catheter ablation" OR "Pulmonary vein isolation"		3
Google Scholar	"BMP-10" OR "Bone morphogenetic protein 10" AND "Atrial fibrillation recurrence" AND "Catheter ablation"	Last 10 years	5

BMP-10: bone morphogenetic protein-10; MeSH: Medical Subject Headings.

### Inclusion and exclusion criteria

The studies were included if they enrolled AF patients undergoing catheter ablation, measured BMP10, reported AF recurrence, and provided hazard ratios (HRs). Studies on PubMed/Medline, Cochrane Library, and Europe PubMed Central from inception through February 2026, and in last 10 years on other databases as mentioned above were included. The eligible studies were published in English, focused on human subjects, and included prospective cohort studies evaluating BMP10 association with post-ablation AF recurrence.

Studies were excluded if they were published more than 10 years ago on the databases other than PubMed/Medline, Cochrane Library, and Europe PubMed Central, written in languages other than English, or based on non-human research to minimize bias and maintain the review’s focus. Additionally, studies with incomplete data, conference abstracts, case reports, editorials, posters, preprints, presentations, and non-peer reviewed studies were excluded.

### Selection process

All identified records were imported into EndNote (Clarivate, Philadelphia, PA), and duplicates were removed. Two independent reviewers screened titles and abstracts for eligibility based on predefined inclusion and exclusion criteria. Full-text articles of potentially eligible studies were then assessed independently by the same two reviewers. Disagreements at any stage were resolved through discussion and consensus; if consensus could not be reached, a third senior reviewer adjudicated.

### Quality appraisal and data extraction

Quality appraisal was conducted independently by two reviewers with prior training in systematic review methodology. These observational studies were assessed using Newcastle–Ottawa Scale (NOS). Inter-reviewer agreement for title/abstract screening and full-text eligibility assessment was quantified using Cohen’s kappa (κ). Agreement was substantial (κ = 0.82) for study selection and moderate-to-substantial (κ = 0.76) for quality appraisal. Discrepancies were resolved through discussion and consensus, with arbitration by a third reviewer when necessary. NOS scores were categorized as high quality (≥ 7), moderate quality (5–6), and low quality (< 5). Methodological quality was assessed to evaluate the possibility of bias and overall strength of the available evidence. Study quality did not determine eligibility for inclusion or exclusion; rather, NOS assessments were utilized to inform the interpretation of the findings, with greater focus placed on data drawn from studies of better methodological quality.

Data extraction was performed independently by one reviewer and verified by a second reviewer to ensure accuracy and completeness. Data extraction included: study title, first author, publication year, study design, sample size, mean age, sex distribution, ablation strategy, follow-up duration, rhythm monitoring modality, recurrence rate, HR, confidence intervals (CIs), and covariate adjustments.

### Outcomes and follow-up

The primary outcome was late AF recurrence, defined as any documented atrial arrhythmia (AF, atrial flutter, or atrial tachycardia lasting ≥ 30 s) occurring after a 3-month post-ablation blanking period, in accordance with contemporary consensus definitions. Early arrhythmia occurring within the first 3 months post ablation was classified as part of the blanking period and was not considered recurrence. The primary exposure of interest was pre-ablation BMP10 level. Rhythm monitoring was performed using 12-lead electrocardiogram (ECG), 24-h ECG monitoring, and 7-day Holter monitoring at scheduled follow-up intervals (typically 1, 3, 6, and 12 months or longer).

### Statistical analysis

The meta-analysis was performed using Review Manager (RevMan) 5.4.1. HRs were pooled using a random-effects generic inverse-variance meta-analysis due to expected clinical and methodological variability. Effect estimates (HRs) were log-transformed prior to pooling, and standard errors were calculated from the 95% CIs (Supplementary Material 1, jocmr.elmerjournals.com).

The primary analysis pooled unadjusted HRs based on continuous BMP10 levels across all studies using a random-effects model. Secondary analyses included: (1) adjusted HRs from studies reporting continuous BMP10 levels; and (2) adjusted HRs from all studies in a secondary exploratory analysis that incorporated all effect estimates, including one study reporting HRs per quartile increase in BMP10. Because continuous and quartile-based HRs are estimated on different exposure scales, this analysis was performed solely to evaluate the consistency of the direction of association across adjusted studies and should not be interpreted as a precise quantitative pooled effect estimate.

All analyses followed Cochrane Handbook recommendations for time-to-event outcomes. Heterogeneity was assessed using the I^2^ statistics, with cutoffs of 25%, 50%, and 75% defining low, moderate, and high heterogeneity. Publication bias was not assessed due to the inclusion of fewer than 10 studies. A P-value of < 0.05 was considered statistically significant for all analysis.

## Results

The initial number of relevant studies identified on databases was 30. Endnote, in total, removed seven duplicated articles. We reviewed 23 articles in detail, screened through titles and abstracts, and assessed for full text availability. As a result, nine articles were shortlisted for retrieval and 14 papers were excluded. Shortlisted studies were evaluated using inclusion, exclusion criteria, and quality appraisal tools, and six articles were removed. As a result, three relevant articles were included in the meta-analysis. The PRISMA flowchart outlining the screening and selection process is shown in Figure 1.

**Figure 1:**
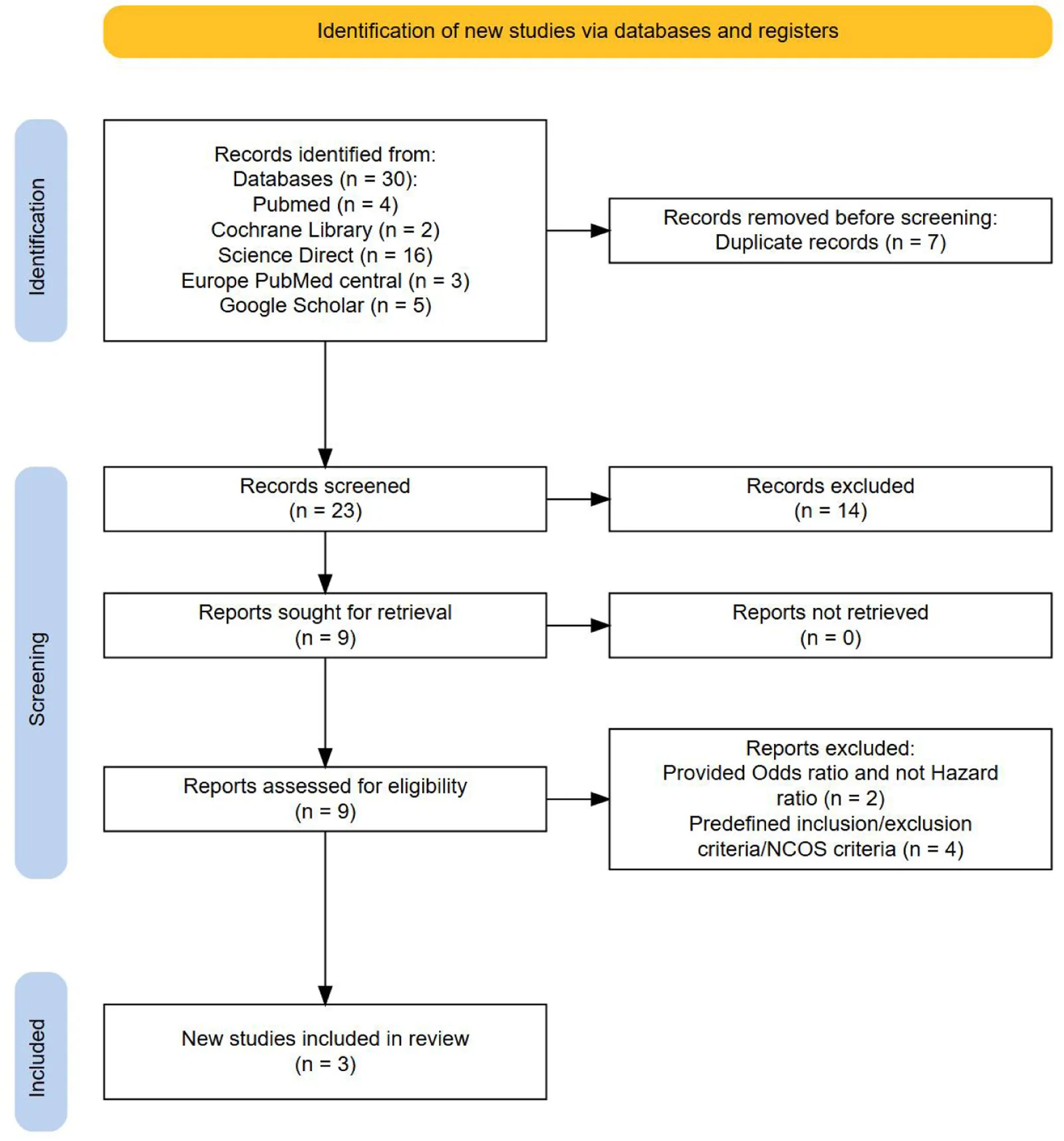
PRISMA flow diagram.

### Study characteristics

A total of three studies were included in the quantitative synthesis, comprising patients undergoing catheter ablation for AF. The largest prospective cohort study done in Switzerland had 1,112 participants while other two prospective cohorts were multi-centric and encompassed 433 and 359 participants. The key findings of these studies are summarized in Table 2 [20, 25, 26].

**Table 2:** Study Characteristics

Study details, country	Study design	Ablation strategy	AF recurrence (%)	Mean/median follow-up	Sample size	Male (%)	Mean age (years)	BMI (kg/m^2^)	BMP10 and AF recurrence, HR (95% CI)
Chua et al, 2024, International/multi-centric [25]	Prospective biomarker cohort analysis of AXAFA-AFNET5 trial	First time catheter ablation (PVI + other)	120 (28%)	3 months (ECG and 24-h ECG monitoring)	433	67%	64	28 (recurrent 29)	Unadjusted HR 1.23 (1.03–1.48) (continuous BMP10) aHR 1.24 (1.02–1.51) (continuous BMP10)
Reyat et al 2020, Multicenter Europe Cohort [20]	Prospective cohort analyses embedded within multiple cohorts (AFACT, MARK AF & AFLMU)	Catheter-based AF ablation AFLMU cohort	153 (42.62%)	12 months (7-day Holter)	359	59.3%	68 (recurrent AF group)	23.3 (recurrent AF group)	Unadjusted HR 1.527 (1.150–2.028) (continuous BMP) aHR (multivariate) 1.339 (1.159–1.546) (per quartile BMP10 increase) aHR (univariate adjustment for age/sex/AF type/left atrial diameter) 1.334 (1.142–1.558) (per quartile BMP10 increase)
Hennings et al 2023, Switzerland [26]	Prospective Cohort (Swiss-AF-PVI registry)	First elective catheter ablation (PVI with RF or cryoballoon)	374 (33.6%)	12 months (ECG + 7-day Holter)	1,112	74%	61	27.3	Unadjusted HR 2.28 (1.43–3.62) (continuous log-BMP10) Age- & sex-adjusted HR 2.08 (1.23–3.52) (continuous BMP10) Multivariable adjusted HR 1.98 (1.14–3.42) (continuous BMP10) Quartile 4 vs. quartile 1, aHR (multivariable) 1.46 (1.06–2.02) (multivariable adjustments for age/sex/BMI/CAD/HTN/HF/diabetes/stroke/renal failure)

AF: atrial fibrillation; aHR: adjusted hazard ratio; BMI: body mass index; CI: confidence interval; ECG: electrocardiogram; HR: hazard ratio; PVI: pulmonary vein isolation; RFCA: radiofrequency catheter ablation.

The mean age of participants across studies ranged from 61 to 68 years, with a predominance of male participants and mixed AF subtypes (paroxysmal and persistent). The follow-up duration varied from 3 to 12 months, and most patients underwent first-time catheter ablation. The AF recurrence was defined as documented episodes ≥ 30 s on follow-up ECG or Holter monitoring. The AF recurrence rates varied from 28 % to 42.62% across studies.

### Quantitative synthesis

#### Primary analysis (unadjusted HRs, continuous BMP10)

All three studies contributed unadjusted HRs based on continuous BMP10 measurements. In the largest Swiss-AF-PVI cohort, a per-unit log-transformed BMP10 was associated with a significantly higher risk of recurrence (HR 2.28, 95% CI 1.43–3.62; P < 0.0001).

Pooled analysis demonstrated a statistically significant association between higher BMP10 levels and increased risk of AF recurrence following catheter ablation as shown in Figure 2 (pooled HR 1.53, 95% CI 1.13–2.07, I^2^ = 69%, P = 0.006).

**Figure 2:**
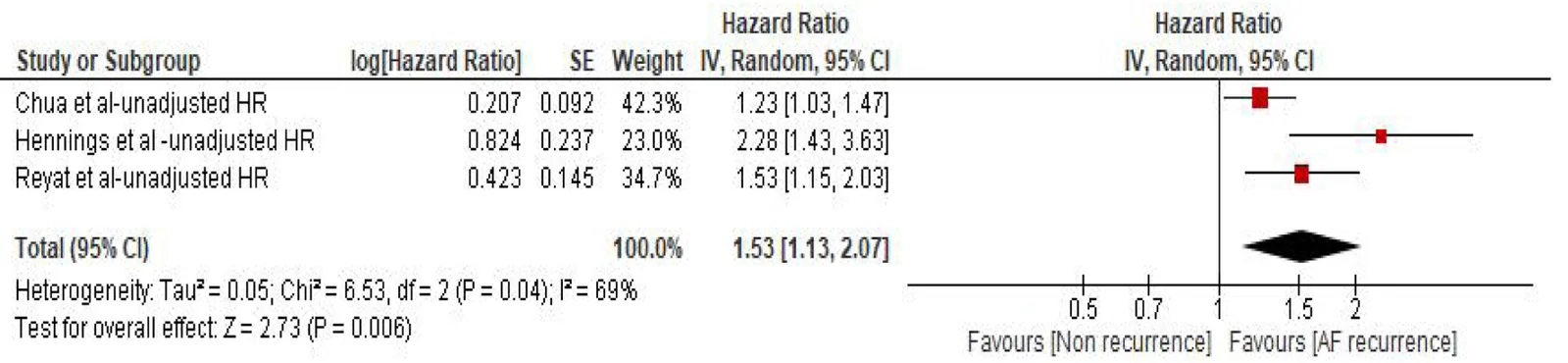
Forest plot illustrating random-effects meta-analysis of unadjusted HR of BMP10 association with post-ablation AF recurrence. AF: atrial fibrillation; BMP10: bone morphogenetic protein-10; HR: hazard ratio.

The consistent direction of effect was observed across studies, with higher BMP10 levels being uniformly associated with an increased risk of recurrence, thus supporting the robustness of the pooled estimate.

#### Adjusted analysis (continuous BMP10, two studies)

Two studies reported multivariable-adjusted HRs using continuous BMP10 levels. Adjustment models included demographic variables, AF type, and cardiovascular comorbidities. Both individual studies demonstrated statistically significant correlations between higher BMP10 levels and recurrence of AF. However, when these estimates were pooled, the overall association was attenuated and did not reach statistical significance as shown in Figure 3 (pooled HR 1.46, 95% CI 0.94–2.25; P = 0.09; I^2^ = 59%), indicating that the observed association should be interpreted with caution.

**Figure 3:**
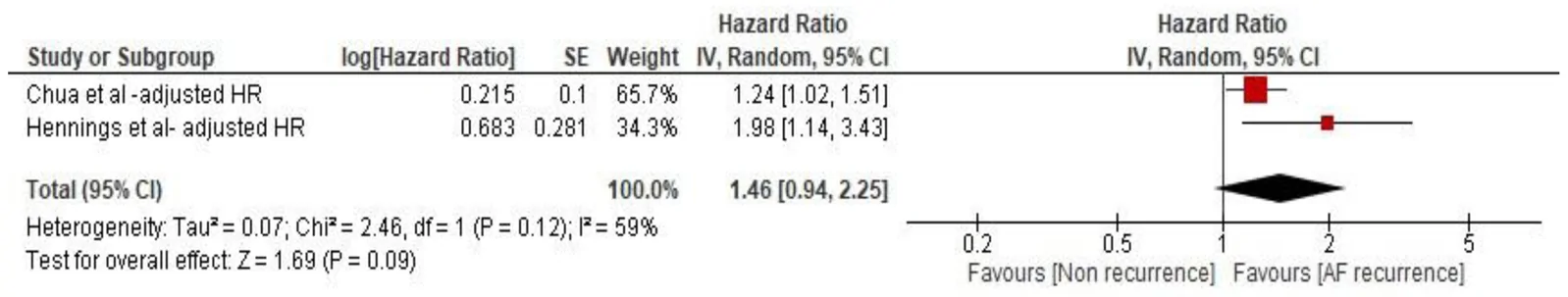
Forest plot illustrating random-effects meta-analysis of adjusted HR of BMP10 association with post-ablation AF recurrence. AF: atrial fibrillation; BMP10: bone morphogenetic protein-10; HR: hazard ratio.

#### Exploratory analysis (all studies, mixed scaling)

In the Swiss-AF-PVI cohort, patients in the highest quartile had significantly higher risk of AF compared to patients in the lowest quartile (adjusted HR 1.46, 95% CI 1.06–2.02) with a significant linear trend across the quartiles (P = 0.02).

In the AFLMU cohort study, the risk of recurrence increased significantly with each quartile increase in BMP10 (adjusted HR 1.34), in line with a graded relationship between biomarker levels and clinical outcomes.

In the third cohort study, increasing BMP10 levels were associated with progressively increased risk of AF recurrence (adjusted HR 1.24).

An exploratory analysis incorporating all adjusted effect estimates of all three studies, irrespective of reporting scale (continuous versus quartile-based), demonstrated a consistent direction of association across studies (pooled HR 1.33, 95% CI 1.16–1.53, I^2^ = 20%, P <0.0001) as shown in Figure 4. However, given that the effect estimates were derived from different exposure scales, the pooled estimate should be cautiously interpreted as an assessment of consistency rather than as a precise quantitative summary measure.

**Figure 4:**
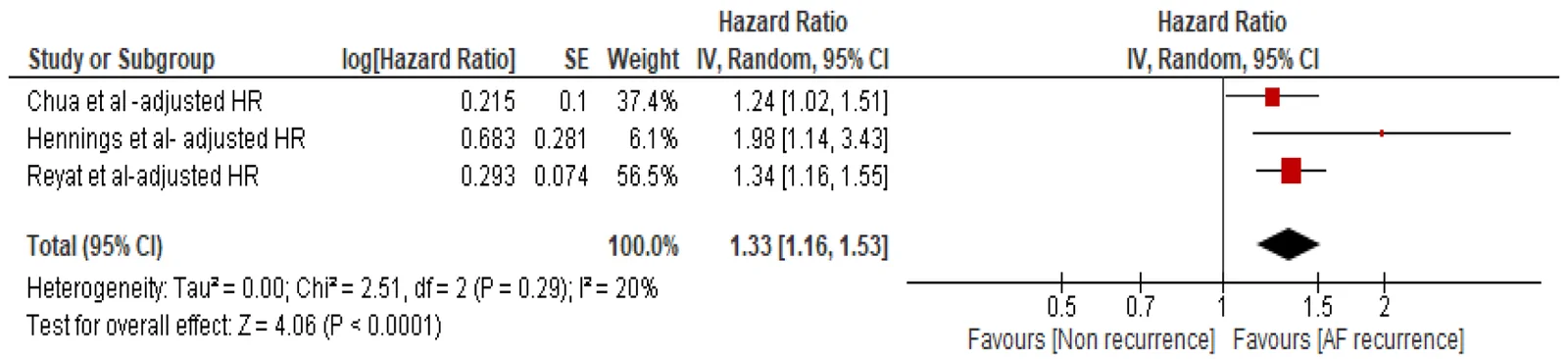
Forest plot illustrating exploratory analysis of BMP10 (mixed scaling) association with post-ablation AF recurrence. AF: atrial fibrillation; BMP10: bone morphogenetic protein-10.

## Discussion

This meta-analysis is the first synthesized evidence to our knowledge to evaluate BMP10 as a biomarker for predicting AF recurrence after catheter ablation. The primary finding of this meta-analysis suggests that circulating BMP10 is a promising biomarker associated with the risk of AF recurrence after catheter ablation. However, because the current evidence is drawn from only three prospective studies with methodological heterogeneity, these findings should be interpreted cautiously and considered hypothesis-generating until validated by larger prospective studies.

The primary analysis (unadjusted HRs using continuous BMP10 levels in three studies) was significantly associated with a pooled HR of 1.53 (95% CI 1.13–2.07; P = 0.006), indicating an approximately 50% higher AF recurrence risk with increasing BMP10 levels. However, this estimate was accompanied by a high heterogeneity (I^2^ = 69%), indicating variability of effect size across studies and populations.

The moderate-to-high heterogeneity seen in the primary analysis (I^2^ = 69%) should be cautiously interpreted because only three studies were available, hence limiting the precision of statistical heterogeneity estimates and the power of heterogeneity tests. Nevertheless, multiple clinically plausible reasons of between-study heterogeneity exist. These include difference in patient characteristics, proportions of paroxysmal and persistent AF, first-time versus elective ablation procedures, ablation techniques (including radiofrequency and cryoballoon approaches with variable adjunctive lesion sets), follow-up duration (3 to 12 months), and rhythm monitoring strategies (24-h ECG monitoring versus 7-day Holter monitoring). Additional heterogeneity may have arisen from variations in BMP10 assay methods and statistical modeling of biomarker exposure. Because of the small number of studies, subgroup analyses and meta-regression were not practicable; hence, the specific contribution of each factor to the observed heterogeneity could not be formally assessed.

Although both individual studies showed statistically significant adjusted associations when adjusted for other factors, the combined analysis of continuous BMP10 levels did not reach statistical significance (pooled HR 1.46, 95% CI 0.94–2.25; P = 0.09). This result aligns with findings from a large cohort study, which found that the link between BMP10 and AF recurrence became attenuated after adjusting for other factors [26]. The attenuated pooled association after considering multiple variables suggests that the predictive value of BMP10 is partly influenced by its connection to well-established determinants of AF recurrence, including atrial structural remodeling, atrial enlargement, presence of comorbidities, and other markers of atrial cardiomyopathy. Therefore, BMP10 should not be interpreted as a standalone predictor of AF recurrence but instead, it is more appropriately viewed as a circulating surrogate biomarker reflecting the underlying severity of atrial disease. This interpretation is biologically consistent with the atrial-specific expression of BMP10, a cardiomyocyte-derived protein belonging to the transforming growth factor-β superfamily, and its regulation by transcription factor PITX2, a key determinant of atrial electrophysiological and structural integrity [27]. Therefore, BMP10 might offer additional prognostic information by identifying patients with more severe atrial cardiomyopathy who are naturally at higher risk of AF recurrence following catheter ablation, rather than acting as an independent causal determinant of recurrence.

Nevertheless, the exploratory analysis combining all three adjusted estimates, including one quartile-based study [20], demonstrated a statistically significant overall association (HR 1.33, 95% CI 1.16–1.53; P < 0.0001) with low heterogeneity (I^2^ = 20%). Since this was an exploratory analysis that mixed effect estimates from different types of exposure measures (continuous and quartile-based BMP10), the results should be interpreted as demonstrating consistency in the direction of association rather than providing an exact quantitative pooled estimate. Overall, these results suggest that BMP10 might offer an incremental predictive value. However, more research in larger prospective studies with consistent and standardized reporting of effect estimates is needed before any strong conclusions can be drawn.

In a mechanistic clinical study, elevated BMP10 levels—reflecting PITX2 repression—were independently associated with AF recurrence after ablation, with an adjusted HR of approximately 1.34 per quartile increase [20]. These findings support the concept that BMP10 serves as a surrogate marker of atrial cardiomyopathy, encompassing fibrosis, electrical remodeling, and impaired atrial contractility—substrates that predispose to recurrence despite pulmonary vein isolation.

The clinical relevance of BMP10 is further supported by large prospective cohort data demonstrating its strong and independent association with AF recurrence after catheter ablation. In a cohort of more than 1,000 patients, high BMP10 levels at baseline were significantly associated with AF recurrence both in unadjusted (HR 2.28) and adjusted (HR 1.98) analyses, with a clear dose–response relationship [26]. Furthermore, BMP10 has been shown to predict AF recurrence better than several classical traditional cardiovascular biomarkers, indicating greater specificity for atrial pathology [20]. Apart from recurrence, BMP10 has been associated with broader AF-related adverse events such as stroke, heart failure, and death, lending further weight to its role as a marker of the severity of atrial disease [28].

BMP10 has the advantage of being atrial-specific compared to traditional biomarkers such as natriuretic peptides or inflammatory markers. Natriuretic peptides are markers of hemodynamic stress and volume overload, whereas BMP10 appears to be a more direct marker of atrial structural and molecular remodeling. Such specificity may explain the consistent association with AF-related outcomes in several studies. Nevertheless, recent genetic and Mendelian randomization data indicate that the BMP10–AF association may not be purely causal, but rather may reflect underlying disease processes [29]. This difference is relevant when BMP10 is considered as a prognostic marker rather than a therapeutic target.

The present meta-analysis results provide consistent evidence for BMP10 as a clinically relevant biomarker for the prediction of AF recurrence after catheter ablation. Agreement of unadjusted and adjusted analyses, albeit with a diminished effect size, suggests that BMP10 is a marker of underlying atrial disease severity, rather than just a marker of confounding associations. These findings indicate that BMP10 could be a useful biomarker for identifying atrial cardiomyopathy and assessing the severity of the disease. The most significant benefit of BMP10 may come from using it in combination with other multimodal prediction models rather than being used in isolation as a part of risk assessment strategy. Additionally, the current evidence does not support using BMP10 as a standalone predictor for AF recurrence after ablation. The added value of BMP10 in predicting outcomes beyond existing clinical and imaging models needs to be validated through larger prospective studies.

### Limitations

Several limitations of this meta-analysis should be noted. First, the number of included studies was small, which limited statistical power and evaluation of between-study heterogeneity, and prevented formal evaluation of publication bias. Second, the heterogeneity of reporting in biomarkers either as HRs as a continuous or by quartile leads to methodological inconsistency and the adjusted effect estimates were reported using different exposure scales (continuous versus quartile-based BMP10), precluding direct comparability and limiting the interpretation of the exploratory mixed-scale pooled analysis. Third, the observational nature of included studies may have led to residual confounding in adjusted analyses. Fourth, there is variability in BMP10 assay methods and no standardized thresholds for immediate clinical applicability. In the end, differences in follow-up duration and rhythm monitoring strategies across studies may influence the rates of recurrence detection.

### Conclusion

This meta-analysis suggests that higher circulating BMP10 levels may be associated with an increased risk of AF recurrence following catheter ablation. The unadjusted analyses showed a stronger association and higher heterogeneity; however, adjusted models demonstrated a modest but consistent independent effect. Nevertheless, the evidence is based on a limited number of prospective studies and should therefore be interpreted with caution. BMP10 is a biologically plausible and clinically promising biomarker of atrial remodeling, but larger multicenter prospective studies with standardized biomarker assessment are needed to validate the independent prognostic value of BMP10 before its incorporation into routine clinical risk stratification.

## Supplementary Material

Suppl 1 Supplementary methods: consistent with Cochrane Handbook recommendations of generic inverse-variance meta-analysis.

## Data Availability

The authors declare that data supporting the findings of this study are available within the article and its supplementary information files.
