# Supplementary material for: Bone Morphogenetic Protein-10 as a Predictor of Atrial Fibrillation Recurrence After Catheter Ablation: A Systematic Review and Meta-Analysis
Source: J Clin Med Res. 2026 Sep 26;18(9):643–51. doi: 10.14740/jocmr6640 (PMC13661091; doi:10.14740/jocmr6640)
Supplement: Supplementary file 1 [file jocmr-18-09-643-s001.docx]

**Suppl 1.** Supplementary methods: consistent with Cochrane Handbook recommendations of generic inverse-variance meta-analysis.

| **Studies** | **log(HR)** | **SE** | **HR** | **95% CI** |
| --- | --- | --- | --- | --- |
| Chua et al. 2024 (25) | 0.207  0.215 | 0.092  0.100 | HR 1.23 (Continuous BMP10)  aHR 1.24 (Continuous BMP10) | 1.03–1.48  1.02–1.51 |
| Reyat et al. 2020 (20) | 0.423  0.293 | 0.145  0.074 | HR 1.527 (Continuous BMP10)  aHR1.34 per quartile BMP10 | 1.150–2.028  1.16–1.55 |
| Hennings et al. 2023 (26) | 0.824  0.683 | 0.237  0.281 | HR 2.28 (Continuous BMP10)  aHR 1.98 (Continuous BMP10) | 1.43–3.62  1.14–3.42 |
| Standard Error (SE), $SE=\frac{ln (Upper CI) - ln (Lower CI)}{2 x 1.96}$  Log (HR) = ln (HR), ln = natural log, HR = Hazard ratio, CI = Confidence interval, aHR =adjusted hazard ratio | | | | |
